# Interventional treatment of giant tracheal lymphoma under rigid bronchoscopy: A case report and literature review

**DOI:** 10.1097/MD.0000000000036736

**Published:** 2024-01-12

**Authors:** Longju Zhang, Jiao Yang, Ju Wang, Jiahao Wu, Sulu Shen, Chuwei Deng

**Affiliations:** a Department of Respiratory and Critical Care Medicine, The Third Affiliated Hospital of Zunyi Medical University/The First People’s Hospital of Zunyi, Zunyi, China; b Pathology Department, The Third Affiliated Hospital of Zunyi Medical University/The First People’s Hospital of Zunyi, Zunyi, China.

**Keywords:** bronchoscopy, interventional therapy, tracheal lymphoma

## Abstract

**Introduction:**

Lymphoma can appear in all parts of the body and present with different symptoms. However, bronchial lymphoma is rare and can be misdiagnosed as airway malignancy or lung disease.

Patient: An older adult woman with tracheal lymphoma experienced severe breathing difficulties, and chest computed tomography indicated severe narrowing of the airway. She did not respond to repeated antibiotic treatment, and she was eventually diagnosed with lymphoma based on pathology after surgical removal of the tumor.

**Diagnosis:**

The patient received a diagnosis of thoracic tracheal stenosis due to intratracheal inflammatory granulomatous lesions or a tumor.

**Interventions:**

Treatment involved the use of a high-frequency electrotome, freezing, and argon plasma coagulation.

**Outcomes:**

The patient reported improvements in dyspnea, cough, and other symptoms after the operation. The pathological results confirmed follicular lymphoma. Reexamination by fiberbronchoscopy indicated that the degree of stenosis in the middle and upper tracheal segments was significantly reduced following interventional therapy.

**Conclusion:**

Endoscopic interventional therapy can be an effective treatment for tracheal lymphoma.

## 1. Introduction

Lymphomas commonly lead to widespread lymph node enlargement, bone marrow infiltration, and an enlarged spleen. Primary lesions are not typically found in the trachea and can easily be misdiagnosed as a bronchogenic carcinoma or a benign tracheobronchial tumor.^[1]^ In this case report, we present a 77-year-old woman with follicular lymphoma (FL) involving the trachea, who initially presented with a cough and dyspnea. Additionally, we reviewed the case reports of 11 patients who underwent interventional therapy for tracheal lymphoma.^[2–8]^

## 2. Case report

### 2.1. Patient information

A 77-year-old Chinese woman had a prolonged history of biofuel exposure. On March 14, 2018, she was diagnosed with chronic obstructive pulmonary disease and stenosis in the upper segment of the trachea, which was observed on chest computed tomography (CT). Since the patient did not experience significant dyspnea or discomfort, no specific treatment was provided at that time. However, she later developed a persistent cough and increased shortness of breath, particularly during physical activities. By June 20, 2018, the patient experienced significant dyspnea (mainly during the inspiratory phase) and was immediately admitted to hospital. Her vital signs were within the normal range, but her oxygen saturation levels were slightly low (SPO_2_, 94% [without oxygen inhalation]). This article has obtained the oral informed consent of the patient family to publish the patient anonymous information in the article.

### 2.2. Clinical findings

**Figure 1.**
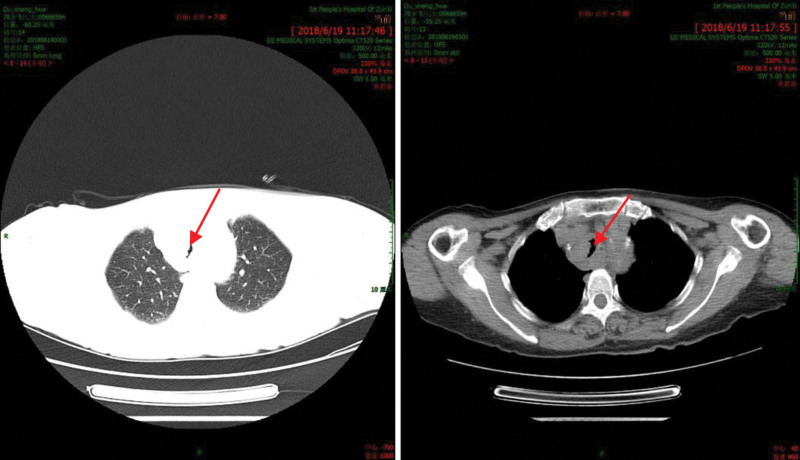
Computed tomography (CT) scan of the chest indicating soft-tissue lumen eminence and tracheal stenosis in the thoracic segment of the trachea.

Upon examination, there were no signs of the 3-concave deformity during breathing, mouth ulcers, enlarged superficial lymph nodes, weakened tactile fremitus in both lungs, hyperresonance upon percussion in both lungs, coarse breath sounds in both lungs, or scattered wheezing, with only slight moist rales in both lower lungs. Cardiac and abdominal examinations revealed no apparent abnormalities. Blood gas analysis showed a low oxygen partial pressure (pH, 7.440; PaCO_2_; 34.3 mm Hg; PO_2_, 69.7 mm Hg; BE, 0.3 mmol/L; HCO_3_, 23.5 mmol/L). CT revealed a soft-tissue-like protrusion within the thoracic segment of the trachea, causing narrowing of the airway. This finding was suggestive of an inflammatory granulomatous lesion (Fig. 1). All other laboratory tests yielded normal results. The patient had been previously diagnosed with hypertension, hypertensive heart disease, cerebral infarction, and Alzheimer disease. She reported no history of smoking or drinking.

### 2.3. Therapeutic intervention

The patient was admitted to the hospital and treated with antibiotics, bronchodilators, and anti-inflammatory agents. However, conventional treatment was ineffective. Therefore, we resected the airway mass using bronchoscopy. The newly formed organisms protruding from the lumen were first excised with high-frequency electrocautery. The tissue was then frozen and cut. Hemostasis was achieved with an argon knife. The organism was completely resected, and the degree of tracheal stenosis significantly improved.

### 2.4. Diagnostic assessment

**Figure 2.**
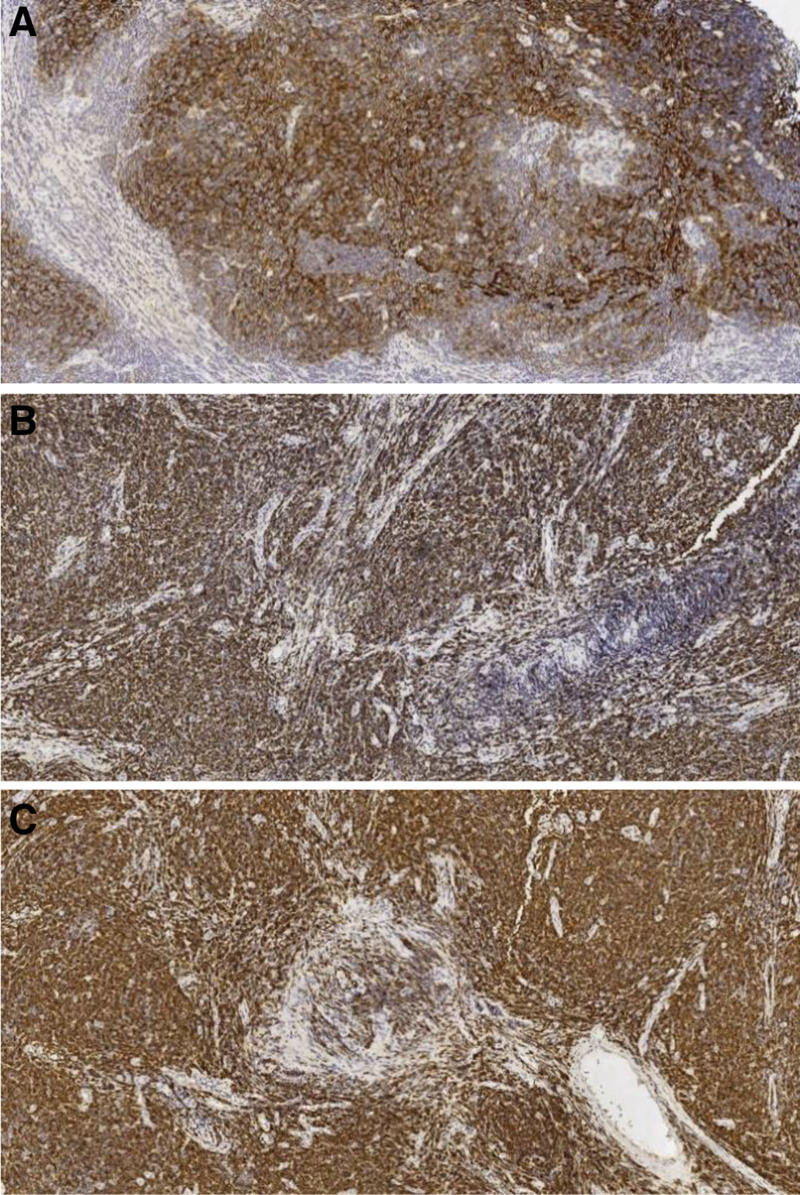
Immunohistochemical staining: (A) CD20 staining of tumor cells was positive (EnVision method × 100 times); (B) BCL-2 staining of tumor cells was positive (EnVision method × 100 times); (C) CD79α staining of tumor cells was positive (EnVision method × 100 times).

Immunohistochemical staining showed positive expression of CD20, Bcl-2, and Ki67, and negative expression of CD3, CD5, and cyclin D1 (Fig. 2), consistent with FL. The frozen tissue submitted for examination was proliferative lymphoid tissue in a state of nodular hyperplasia. The cells were consistent and most of the normal structures were absent. Neoplastic lymphoma lesions could not be ruled out. A pathological diagnosis was sent for examination (main bronchial neoplasm), which was identified as grade I FL.

### 2.5. Follow-up and outcomes

**Figure 3.**
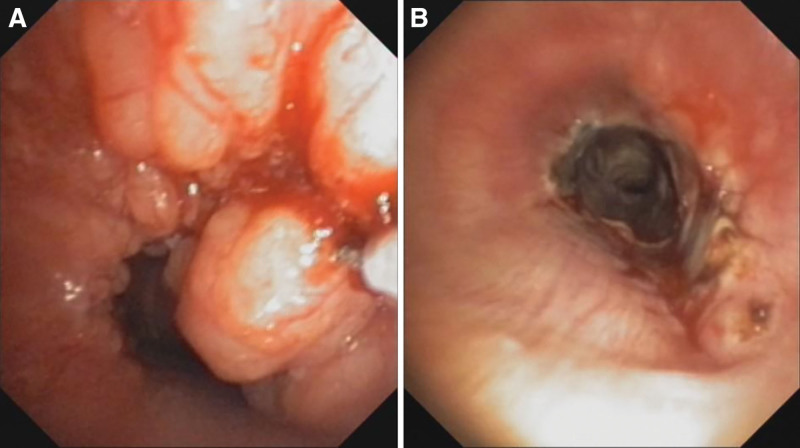
Bronchoscopy results: (A) The trachea was largely obstructed by a nodular mass. (B) Bronchoscopic findings after interventional therapy.

Postoperatively, the patient felt that the symptoms of dyspnea were significantly reduced. Her vital signs were good. Repeated fiber optic bronchoscopy (Fig. 3) indicated that after interventional treatment of the neoplasms in the upper and middle trachea, the degree of stenosis was significantly reduced, and the surface was covered with yellow–white necrotic substances. The timeline of diagnoses and interventions are shown in Table 1.

**Table 1:** Timeline of diagnoses and interventions.

Time	Symptoms	Examination	Intervention
2018.3.14	–	Computed tomography	–
2018.6.20	Breathing difficulties	Computed tomography	Anti-infection
2018.6.23	Worse	–	Interventional therapy
2018.6.26	Better	Fiber optic bronchoscopy	–

## 3. Discussion

We reviewed the medical records of 12 patients with tracheal lymphomas who were treated with bronchoscopic interventions (Table 2). To our knowledge, this is the largest series of patients with tracheobronchial lymphoma treated with bronchoscopy. The patients’ ages ranged from 15 to 77 years (average: 47 years). The most common clinical symptoms were dyspnea (10/12, 83.33%) and cough (4/12, 33.33%). The most common histological subtypes were mucosa-associated lymphoid tissue lymphoma (3/12, 25%) and anaplastic large cell lymphoma (3/12, 25%), which is consistent with other reports. The most commonly used interventional therapy was stent placement (7/12, 58.33%). Among them, 3 patients also received chemotherapy after surgery. There were no treatment complications except for 1 patient who developed pulmonary thromboembolism on day 7 after surgery and 1 patient who had stent dislocation. Most patients had a good prognosis after interventional therapy; except for 1 patient who died in the 7th month after diagnosis due to insensitivity to subsequent chemotherapy drugs. The remaining patients showed no signs of recurrence during the follow-up period, and their respiratory symptoms were significantly relieved.

**Table 2:** Information on the 12 patients with tracheal lymphoma treated by bronchoscopic intervention.

Serial number	Age	Gender	Cardinal symptom	Physical sign	Degree of coronary artery stenosis	Therapeutic method	Complications	Pathology	Follow-up
1	15	Male	Dyspnea, hemoptysis	Fever, subcutaneous emphysema of shoulder and chest	–	Stent + chemotherapy	–	ALCL	7 mo
2	54	Female	Dyspnea, dry cough, wheezing	–	Trachea 80%	HFES	–	NSCHL	–
3	60	Female	Dry cough, wheezing, hoarseness	–	Trachea 80%	Nd-YAG laser + radiotherapy	–	MALT lymphoma	–
4	74	Female	–	–	Midtracheal 10 mm tumor	Nd-YAG laser + IEI	–	MALT lymphoma	12 mo
5	49	Female	Breathing difficulties	Sound of breath is rough	–	Stent + chemotherapy	–	MALT lymphoma	12 mo
6	56	Male	Difficulty breathing and coughing	Low left lung breathing sound	–	APC + freezing + electrocoagulation + chemotherapy	–	DLBCL	24 mo
7	77	Female	Cough, shortness of breath	Rough breathing, wet rales, and wheezing in both lungs	Trachea 60%	HFES + freeze + APC	–	FL	23 mo
8	37	Female	Breathing difficulties	–	RMB > 90%	Stent	Stent displacement	ALCL;MCL	–
9	64	Female	Breathing difficulties	–	RMB > 90% LMB ˃ 50% Tracheal 50%	Stent	–	NSCHL	–
10	31	Female	Breathing difficulties	–	LMB ˃ 60% Tracheal ˃ 90%	Stent	–	NSCHL	–
11	16	Female	Breathing difficulties	–	RMB ˃ 90% LMB ˃ 90% Tracheal 60%	Stent	–	DLBCL	–
12	31	Male	Breathing difficulties	–	RMB ˃ 80% LMB ˃ 80%	Stent	Pulmonary embolism	ALCL	–

ALCL = anaplastic large cell lymphoma, APC = argon plasma coagulation, DLBCL = diffuse large B-cell lymphoma, FL = follicular lymphoma, HFES = high-frequency electric snare, HL = Hodgkin lymphoma, IEI = intratumoral ethanol injection, LMB = left principal bronchus, MALT lymphoma = extranodal marginal zone lymphoma of mucosa-associated lymphoid tissue, MCL = mantle cell lymphoma, NHL = non-Hodgkin lymphoma, NSCHL = nodular sclerosis classical Hodgkin lymphoma, RMB = right main bronchus.

Primary tracheal lymphomas are rare. The symptoms are not specific, and if the treatment is not effective or the symptoms worsen, CT or bronchoscopy is performed to identify the lymphoma lesions. A histopathological biopsy is the gold standard for diagnosis. As tracheal lymphoma is rare, there are no diagnostic or treatment guidelines. The treatment of most patients is based on the literature or personal experience. Typically, surgery, chemotherapy, and radiotherapy are used.^[9–11]^ Endoscopic interventional technology has advanced rapidly in recent years. Bronchoscopic interventional therapy is a minimally invasive procedure that can be used to quickly and effectively remove endotracheal lesions. It has several advantages, including low operation costs and good patient prognosis. The combined application of multiple interventional therapy technologies can overcome the shortcomings of each individual technique. Therefore, an increasing number of researchers have used combined interventional techniques to treat benign and malignant tracheal stenoses. This patient was treated with high-frequency electrosurgery combined with cryotherapy and argon plasma coagulation. The high-frequency electric knife degenerates and coagulates diseased tissue. Ice crystals produced by cryoprobes when in contact with tissues can damage organelles. Argon plasma coagulation can continuously transmit the coagulation current output from the electrode to the wound surface, resulting in a hemostatic effect. The advantages and disadvantages of the 3 therapies complement each other, effectively relieving patients’ respiratory symptoms, and providing a high level of safety. Some studies have evaluated the safety and efficacy of bronchoscopic interventional therapy for benign and malignant airway tumors, and all have achieved good results.^[12,13]^

In conclusion, tracheal lymphoma is a relatively rare cancer. Its pathogenesis is unknown, and its clinical manifestations are often nonspecific; therefore, it is easily missed or misdiagnosed. Clinicians should be vigilant of patients with recurrent respiratory symptoms and a poor response to conventional treatment. Although there is no guided treatment plan, various treatment modalities have achieved satisfactory results. Compared to conventional radiotherapy and chemotherapy, bronchoscopic interventional therapy is relatively less invasive, has fewer complications, and is an effective treatment method for tracheal lymphoma.

## Acknowledgments

The authors thank all those who contributed to this report and the families of patients who signed informed consent forms.

## Author contributions

**Formal analysis:** Jiahao Wu, Chuwei Deng.

**Investigation:** Sulu Shen.

**Supervision:** Longju Zhang.

**Writing – original draft:** Jiao yang.

**Writing – review & editing:** Ju Wang.

## References

[1] Carbone A Roulland S Gloghini A. Follicular lymphoma. Nat Rev Dis Primers. 2019;5:83. 31831752 10.1038/s41572-019-0132-x

[2] Boujaoude Z Malin J Abouzgheib W. Hodgkin disease of the trachea. J Bronchology Interv Pulmonol. 2012;19:200–2. 23207461 10.1097/LBR.0b013e31825c357d

[3] Ding J Chen Z Shi M. Tracheal stenting for primary tracheal mucosa-associated lymphoid tissue lymphoma. Eur J Med Res. 2013;18:8. 23547898 10.1186/2047-783X-18-8 PMC3621548

[4] Huang IA Hsia SH Wu CT. Combined chemotherapy and tracheobronchial stenting for life-threatening airway obstruction in a child with endobronchial non-Hodgkin lymphoma. Pediatr Hematol Oncol. 2004;21:725–9. 15739628 10.1080/08880010490514930

[5] Mira-Avendano I Cumbo-Nacheli G Parambil J. Mucosa-associated lymphoid tissue lymphoma of the trachea. J Bronchology Interv Pulmonol. 2012;19:44–6. 23207262 10.1097/LBR.0b013e31823fadc2

[6] Schmidt B Massenkeil G John M. Temporary tracheobronchial stenting in malignant lymphoma. Ann Thorac Surg. 1999;67:1448–50. 10355429 10.1016/s0003-4975(99)00254-4

[7] Tsurutani J Kinoshita A Kaida H. Bronchoscopic therapy for mucosa-associated lymphoid tissue lymphoma of the trachea. Intern Med. 1999;38:276–8. 10337941 10.2169/internalmedicine.38.276

[8] Yang FF Gao R Miao Y. Primary tracheobronchial non-Hodgkin lymphoma causing life-threatening airway obstruction: a case report. J Thorac Dis. 2015;7:E667–71. 26793387 10.3978/j.issn.2072-1439.2015.12.05 PMC4703689

[9] Hashemi SMS Heitbrink MA Jiwa M. A patient with endobronchial BALT lymphoma successfully treated with radiotherapy. Respir Med. 2007;101:2227–9. 17616383 10.1016/j.rmed.2006.11.028

[10] Hoskin PJ Kirkwood AA Popova B. 4 Gy versus 24 Gy radiotherapy for patients with indolent lymphoma (FORT): a randomised phase 3 non-inferiority trial. Lancet Oncol. 2014;15:457–63. 24572077 10.1016/S1470-2045(14)70036-1

[11] Zhen CJ Zhang P Bai WW. Mucosa-associated lymphoid tissue lymphoma of the trachea treated with radiotherapy: a case report. World J Clin Cases. 2023;11:1607–14. 36926401 10.12998/wjcc.v11.i7.1607 PMC10011992

[12] Lee BR Oh IJ Lee HS. Usefulness of rigid bronchoscopic intervention using argon plasma coagulation for central airway tumors. Clin Exp Otorhinolaryngol. 2015;8:396–401. 26622961 10.3342/ceo.2015.8.4.396 PMC4661258

[13] Wang H Zhang N Tao M. Application of interventional bronchoscopic therapy in eight pediatric patients with malignant airway tumors. Tumori. 2012;98:581–7. 23235752 10.1177/030089161209800507

